# Genome Network and FANTOM3: Assessing the Complexity of the Transcriptome

**DOI:** 10.1371/journal.pgen.0020063

**Published:** 2006-04-28

**Authors:** Yoshihide Hayashizaki, Piero Carninci

The findings of the FANTOM3/Genome Network project have redefined the landscape of the mammalian transcriptome by introducing an extensive collection of novel cDNAs and millions of sequenced tags corresponding to 5′- and 3′-ends of mRNAs. This issue of *PLoS Genetics* includes a special collection of articles that explore the transcriptome complexity being revealed by work on the FANTOM3 dataset. Besides revealing staggering complexity, analysis of this collection is providing an increasing number of novel mRNA classes, expressed pseudogenes, and bona fide noncoding variants of protein-coding genes. In addition, new types of regulatory logic have emerged, including sense–antisense mechanisms of RNA regulation. This high-resolution cDNA collection and its analysis represent an important world resource for discovery, and demonstrate the value of large-scale transcriptome approaches towards understanding genome function.

## The Era of Transcriptome Technology: From RNA to Function

After the completion of several genome sequences [1,2] the scientific community has been pondering what type of technologies are necessary for understanding the underlying biology of genomes. Two classes of novel technologies, one based on the hybridization of nucleic acids and the other on sequencing products from mRNA libraries, are already affecting the way we understand biological systems. Hybridization-based methods, such as genome tiling arrays [3–5], have some specific advantages: in a single experiment they can give a draft description of the transcriptome, or of genome elements selected by chromatin immunoprecipitation (ChIP) [6]. Although a general picture of the transcriptome can be produced quickly, important details such as transcriptional start sites (TSSs) cannot be accurately identified at single-base resolution, nor can such methods determine the exact exon connections and strand orientation, resulting in incomplete sequence information. Considering that 11% of short exons are not represented in Affymetrix tiling arrays, the overall rate of false negative and false positive exon detections is 20% and 5%, respectively (N. Maeda, S. Kondo, D. Sasaki, and Y. Hayashizaki, unpublished data). Despite these limitations, tiling arrays provide an important picture of the genome output, for example, that 41.5% of mRNA is restricted to nuclei, that there are 10-fold more transcribed sequences than there are annotated genes, and that 44% of the RNA sequences are never polyadenylated [3] and thus are missing from cDNA collections.

Methods based on full-length cDNA sequencing [7,8] are more intensive, but provide a picture at a higher resolution, including full sequence and exon–exon connectivity data [9–12]. Because large-scale full-length cDNA sequencing is expensive, novel mRNA tag technologies that are based on deriving sequence tags from full-length cDNAs have been developed (by the RIKEN group and others [13]). Three new technologies speed up the process of analysis: cap analysis of gene expression (CAGE) [14], gene signature cloning [11], and gene identification of signature [15]. The strategy of sequencing the 5′- and/or 3′-ends of transcripts enables increased throughput and brings transcriptome analysis to a new level. These technologies have been fully exploited in the FANTOM (Functional Annotation of the Mouse) and Genome Network (GN) Projects (see Box 1).

This special issue of *PLoS Genetics* is focused on transcriptome analysis done by GN and FANTOM3, carrying the torch from two recently published studies [11,12]. These studies force a paradigm shift in the understanding of the transcriptome. First, the studies find that 63% of the genome is transcribed from at least one strand (in contrast to the earlier belief that only 2% of the genome is transcribed into protein-coding mRNAs). Second, an unexpected amount of variation was found in alternative splice forms (65% of all transcriptional units [TUs] contain alternatively splicing variants), TSSs (which identify promoters), and polyadenylation sites. The number of TUs is somewhat reduced by the occurrence of gene fusion (exon sharing between neighboring genes), but the final number of TUs is still large (>43,000), because of novel mRNAs. Thus, the complex landscape of the transcriptome is revealed, larger than ever before, and we are left with the daunting task of annotating its function and its usage in specific cells.

## Expanding the Bright Matter of the Transcriptome: More Proteins!

Pulling together FANTOM3 and public datasets, no more than 2,200 completely novel proteins were identified, even when analyzing more than 158,000 cDNAs. More striking is the number of protein varieties discovered: combining all the splice variants gives at least 78,000 different mammalian proteins generated from approximately 20,000 protein-coding TUs [11]. Alignment of cDNA to the genome revealed splicing variations of three, six, or nine nucleotides [16] and identified novel splicing sites and splicing mechanisms. The work of van Nimwegen and colleagues improved the alignment of cDNA on the genome by developing the SPA algorithm, which allows a much finer alignment of cDNA to the genome. This advance allows us to better align the sequences, identify the 5′- and 3′-end mapping positions, and reduce the splicing boundary errors, thus improving the study of alternative splicing [17]. Based on the SPA algorithm, Chern et al. further analyzed the functionality of common small-length splice variations at the splicing site [18]. They identified small splice variations (mainly three nucleotides) that take place in 43.7% of all acceptor sites and 23.7% of all donor sites; these variations derive from stochastic binding of the spliceosome to the neighboring splicing site. It is unclear how these small-length splicing polymorphisms affect protein functions in various tissues, nor do we know the extent of this phenomenon in other organisms. We are used to thinking that genes produce compartment-specific proteins. But a new analysis by Davis et al. shows that alternative splicing causes the produced proteins to have different cellular compartment localizations in more than 8% of TUs [19]; determining in greater detail the cell-type specificity of such isoforms will require additional work. Further review of splicing in kinases and phosphatases found that 69% of these family members show alternative splicing [20], including variants that appear nonfunctional, but that with more careful analysis are revealed to be, respectively, decoy receptors and peptides resembling proteolytic forms still capable of binding extracellular solutes. Although experimental validation is needed, such forms would be considered artifacts if we were not analyzing an extensive and redundant dataset.

An open question in the annotation of the transcriptome is the minimal length of a protein. Although this was previously arbitrarily set at 100 amino acids (aa), there is proof of the existence of shorter proteins. Applying the computer program CRITICA in a novel way, Frith and colleagues identified and experimentally verified a missing fraction of the proteome, which contains more than 3,000 candidate proteins (13% of the total number of proteins) that are shorter than 100 aa but longer than 50 aa, a limit below which CRITICA does not perform well, leaving the question unanswered as to whether there are even shorter unknown peptides [21]. Although many of these short proteins are potentially truncated protein variants, there are at least 1,240 that are truly novel short proteins. A part of all of these transcripts seems to be composed of genuine transcripts that seem to originate from within internal exons of longer transcripts, as described below (P. Carninci, unpublished data).

## Expanding the Dark Matter of the Transcriptome: Noncoding RNAs Require Our Attention

In the past, the total number of genes was debated; early estimates ranged from 28,000 to 120,000 genes, based on expressed sequence tag clustering [22–24]. Today, such a large discrepancy can be at least partly explained by the discovery of the large number of noncoding genes and the variability of TU ends, which, before the genome and full-length cDNA sequences were made available, appeared as distinct entities. Additional knowledge of non-polyadenylated RNA based on tiling array technology can also contribute to the explanation [3]. The novel finding of 23,000 noncoding TUs and their prominent biological role in the regulation of gene expression has dramatically changed the traditional view of proteins as the only bioactive molecules, and emphasizes the need to modernize the central dogma.

To better distinguish between protein-coding and noncoding transcript, the FANTOM project has helped develop various computational tools to distinguish between non-protein-coding RNA and regular protein-coding mRNA. One of these tools is addressed in the current issue [25].

Two other papers further expand previous discoveries regarding the complexity of the transcriptome. Furuno et al. [26] analyzed cDNAs that may be constituted of fragments of larger RNAs that are unclonable because of their large size (such as the large noncoding transcripts *Air* and *Xist*), and that are potentially cloned as 5′–3′ truncated cDNA fragments through internal priming. Their search produced 2,700 large noncoding candidate transcripts, of which a small subset was analyzed, and most of them (66 RNAs) were experimentally verified and found to be true noncoding large RNAs with potential regulatory functions, like *Ube3a* and *Kcnq1,* which were identified in this dataset. As a new world of non-polyadenylated RNA was recently identified and found to compose at least half of the unknown transcriptome [3], the number of true noncoding RNAs with regulatory functions found so far promises to be only the tip of the iceberg.

The biological community has considered pseudogenes to be a fossil testimony of old genes once transcribed or reintegrated into the genome, being silent or phenotypically irrelevant [27–30]. A first indication that this view might be incorrect was the discovery a few years back that pseudogenes can be expressed and regulated through RNA–RNA interaction [31]. In this issue, Frith and colleagues [32] have extended the analysis of noncoding transcript expression and have identified 10,000 full-length cDNAs derived from expressed pseudogenes—constituting approximately 10% of the known transcriptome—half of which are promoted by retrotransposons, or otherwise characterized promoters, and are likely to participate in various regulatory mechanisms. These data suggest that we will need to continue to remain open-minded about the function of expressed pseudogenes as “potogenes” (potential genes) [33] and ncRNAs.

## Biological Significance of Sense–Antisense

Antisense regulation of transcription is one of the many roles of RNA [11] and is one way a network of RNA molecules affects the entire organism's phenotype. Short double-stranded RNA has been found to form regulatory chains at the genome, nucleoprotein, transcriptional, post-transcriptional, and translational level (for review see [34]). The extensive analysis of FANTOM3/GN showed that more than 36,000 sense–antisense (S/AS) pairs were encoded in the mouse transcriptome. These pairs seem to cover a large fraction of genes (72% of the total number of transcripts), including several important genes responsible for human genetic diseases and regulation of important cell functions such as cell cycle arrest and apoptosis [12]. Such prevalence of S/AS is confirmed with large-scale expression SAGE analysis, suggesting that more than 50% of the genes show S/AS transcription [35].

Interestingly, CAGE data showed that there is a preference for transcripts that map head-to-head in the genome, and in particular in the case of nuclear genes [12], suggesting a possible mechanism for transcriptional interference. In this issue, Seno and colleagues present an algorithm they developed to search for the expression of CAGE tags in the genome by looking for regional bias in regions that show significant co-regulation of transcription, including S/AS pairs [36]. By pooling together groups of CAGE libraries for similar groups of tissues (e.g., liver, lung, and macrophages, regardless of their condition), they identified S/AS transcription to be particularly overrepresented in certain loci, which strongly supports the hypothesis that transcriptional interference is a mechanism of transcriptional regulation. The next step regarding these data is to determine whether there is a correlation of cellular localization among co-expressed genes (nuclear versus cytoplasmic) and whether they are co-expressed in the same cells and from the same chromosome, in order to distinguish between a number of potential functional mechanisms.

**Figure 1:**
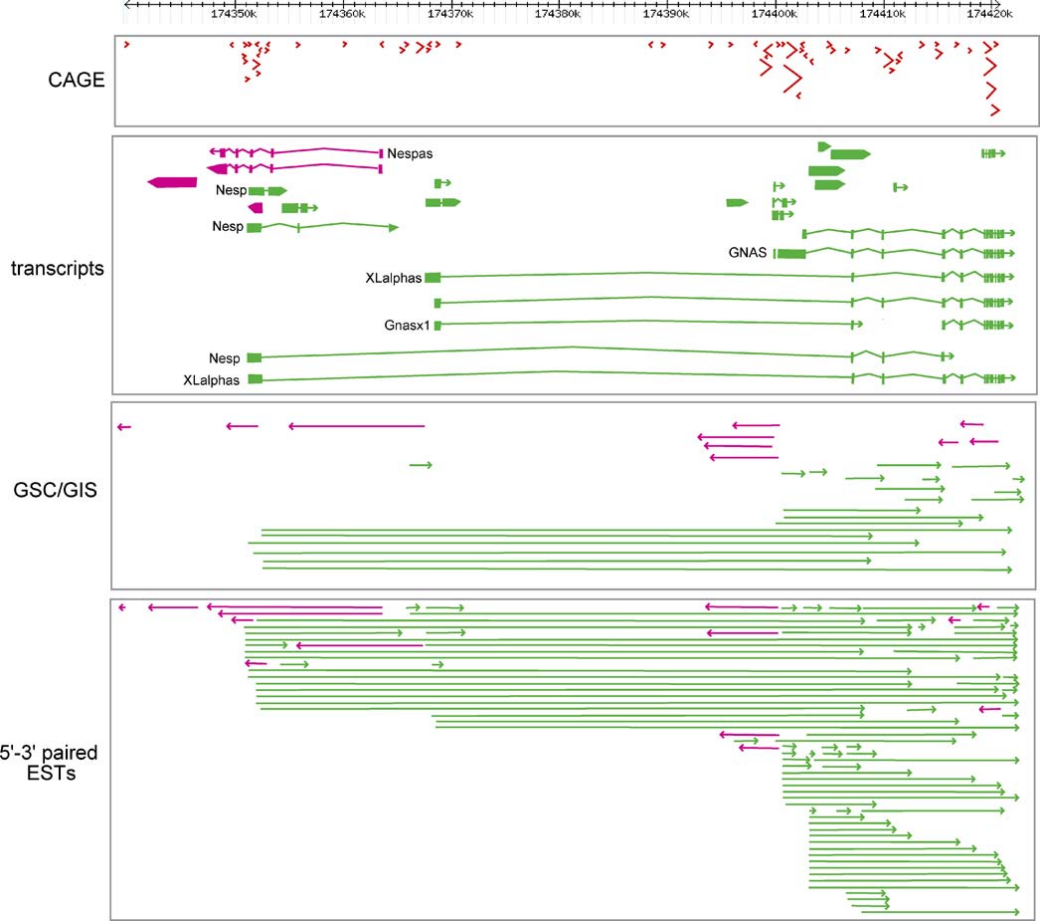
The Imprinted *Gnas* Locus Reveals the Complexity of the Transcriptome There at least 50 different transcripts that overlap into about ten interconnected TUs. The upper panel shows the CAGE tags (TSSs). Pink and green denote the two different transcript orientations on the genome. The size of the arrow corresponds to the number of overlapping CAGE tags. The middle panel shows the regions of the genome that are utilized as exons, and the structure of known transcripts, with their annotation. The bottom two panels show novel transcript boundaries, identified with gene signature cloning and gene identification of signature (GSC/GIS) and 5′–3′ paired expressed sequence tags (ESTs).

The observation that S/AS pairs are frequently clustered in complex genomic regions was made possible by using more than 158,000 cDNAs [11] (Figure 1). Engström et al. made a comprehensive catalog of complex loci, and have define the concept of “chain” for loci including at least three independent transcripts in S/AS relationship to each other or sharing a bidirectional promoter [37]. The rationale for such grouping is that such loci will be subject to common epigenetic relationships. They found that there are approximately 1,000 mouse–human conserved chains (7-fold more than known before) and that these include genes that are overrepresented in cancer, clearly revealing the need for further attention to expression dynamics in gene chains.

## Variability within Transcripts Highlights Complex Transcriptional Regulation Mechanisms

**Figure 2:**
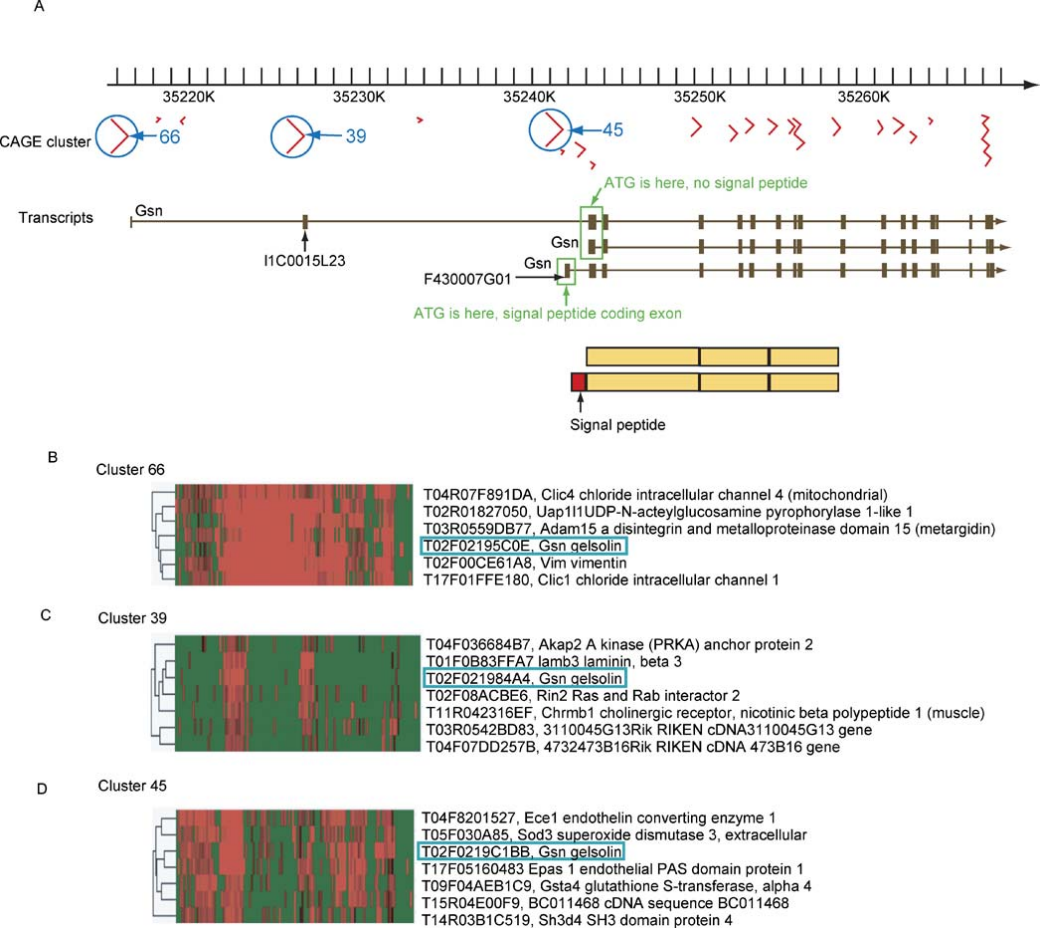
Gene Expression Is Driven by Context: The Mouse Gelsolin Multiple Promoters (A) Map of various isoforms of gelsolin mRNAs on the genome. There are three main TSSs, indicated by blue circles. The numbers are the arbitrary expression cluster numbers (clusters 66, 39, and 45) where these TSSs were assigned. The two upstream promoters produce RNAs that encode for the cytoplasmic isoform, while the third main promoter (cluster 45) encodes an mRNA that produces the secreted isoform of gelsolin. (B–D) The expression context of the three promoters is different. The *x-*axis shows different tissues based on their transcriptional similarity, and the *y-*axis shows TSS clustering. Various forms of gelsolin are expressed in different transcriptional contexts, emphasizing the importance of distinguishing between promoters and isoforms in the expression analysis.

At a first glance, the FANTOM tags dataset has defined TSSs and transcriptional termination sites (TTSs) that exceed the number of TUs, with an average of five different TSSs and TTSs per TU (see Figure 2). Taken together, more than 181,000 different transcripts were identified for cDNAs, with proven evidence of TSSs and TTSs [12]. In particular, the collections of tags have helped in the identification of more than 230,000 TSSs in mouse. CAGE tags alone have identified 160,000 TSSs in mouse and 180,000 TSSs in human [38], expanding the number of well-annotated TSSs by several magnitudes [39]. This dataset has identified a functional dichotomy in mammalian promoters between CpG islands and TATA box promoters. Moreover, these tags have identified further “dark transcription matter” truly originating from 3′ untranslated regions and coding exons. Analysis of expression clusters and their core promoter elements allows identification of regulatory elements and reconstruction of transcriptional networks and subnetworks after the activation of macrophages with lipopolysaccharides [40]. Bajic and colleagues analyzed mouse and human CAGE data and the local sequences around promoters, distinguishing them by their GC content at 5′- and 3′-ends of TSSs. Most of the promoters were indeed enriched in GC content, but there were also groups that were AT-rich in the same regions. The density of transcription factor binding sites and genes classified using Gene Ontology terms [41] highlights the basic differences that underlie transcriptional control of different gene categories, in agreement with our unpublished results. Expanded analysis of larger versions of such datasets will hopefully help not only to identify promoters, but also to predict their possible promoter activity, based on transcription factor binding site mapping and usage.

Taking advantage of CAGE's high resolution, Taylor and colleagues analyzed the evolution of core promoter elements [42]. Of particular interest is the fact that CAGE tags reveal that human CpG-type promoters are under positive evolutionary selection, in contrast to TATA box promoters, which are evolving more slowly because of spacing constraints. As CpG promoters, TSSs are modular and driven by pyrimidine/purine dinucleotides [38]), conferring greater transcription plasticity and thus accelerating evolution, because dramatic biological changes derive not only from protein mutation but also from differential expression levels. It would be interesting to apply CAGE technology to more evolutionarily distant vertebrates and invertebrates and further develop this concept of evolutionary rates as it relates to speciation.

## What Developments Lie Ahead?

Tiling arrays and sequencing-based technologies have provided great insights—identifying several key roles of the RNA and transcription itself, and basic regulatory mechanisms. We have so far uncovered only a portion of the transcriptional complexity that exists, and when considering variation in tissues, cell development, and cell stages, we have barely scraped the surface of the unknown. For future studies, further technological development is needed, such as the recent application of tagging technologies to super-parallel sequencing analysis [43] as a part of the “$1,000 genome project.” We need to be able to screen for the function of noncoding RNA, including the significance of *cis* and *trans* S/AS interactions in living cells, and their roles in transcriptional interference, as epigenetic effectors, and yet unknown roles. Integration of these RNA functions and RNA variability into formally described gene models and their relation with phenotypes will then be required.

These datasets are complementary to and an integral part of the ENCODE project [44], a project devoted to the annotation of the entire human genome's regulatory elements. Integration of these two large-scale datasets will form the framework for future post-genomic studies. Hopefully, the future will see more comprehensive transcriptome projects flanking the basic genome sequencing projects, greatly enriching our knowledge and understanding of biology. 

**Box 1. About the FANTOM and Genome Network Collaboration**

FANTOM and GN (originally a Japanese national project for establishing a system for connecting genes with phenotypes and drug targets/effects by using the same platform in multiple biological systems) collaborated to analyze novel transcriptome data consisting of 103,000 full-length cDNAs, more than 7 million mouse and more than 5 million human CAGE tags, and more than 1 million gene identification of signature/gene signature cloning ditags. This was a global collaboration with many institutes (http://www.mext-life.jp/genome/english/index.html). Within the consortium, three meetings were organized for the annotation of mouse full-length cDNA sequences [9–12].

The strategy of handling the cDNA data is described in Maeda et al. [45]. The data resulting from the annotation meetings are available at http://fantom.gsc.riken.jp, and some integrated genomic data, annotated cDNA, tags, and regulatory data have also been published separately [46,47]. RIKEN cDNA clones will be made available to researchers from DNAFORM (http://www.dnaform.jp/index_e.html). Future plans include a large database, the Genome Network Platform, which will be available at http://genomenetwork.nig.ac.jp/public/english_page.html, led by Gojobori's group at the DNA Data Bank of Japan (http://www.ddbj.nig.ac.jp).

## Abbreviations

aa: amino acids
CAGE: cap analysis of gene expression
GN: Genome Network
S/AS: sense–antisense
TSS: transcriptional start site
TTS: transcriptional termination site
TU: transcriptional unit
